# Metabolic Imaging in Non-Alcoholic Fatty Liver Disease: Applications of Magnetic Resonance Spectroscopy

**DOI:** 10.3390/jcm10040632

**Published:** 2021-02-07

**Authors:** Prarthana Thiagarajan, Stephen J. Bawden, Guruprasad P. Aithal

**Affiliations:** 1 NIHR Nottingham Biomedical Research Centre, Nottingham University Hospitals NHS Trust and the University of Nottingham, Nottingham NG7 2UH, UK; Guru.Aithal@nottingham.ac.uk; 2 Nottingham Digestive Diseases Centre, University of Nottingham, Nottingham NG7 2RD, UK; 3 Sir Peter Mansfield Imaging Centre, School of Physics and Astronomy, University of Nottingham, Nottingham NG7 2QX, UK; stephen.bawden@nottingham.ac.uk

**Keywords:** NAFLD, metabolic liver disease, magnetic resonance, spectroscopy

## Abstract

Non-alcoholic fatty liver disease (NAFLD) is poised to dominate the landscape of clinical hepatology in the 21st century. Its complex, interdependent aetiologies, non-linear disease progression and uncertain natural history have presented great challenges to the development of effective therapies. Progress will require an integrated approach to uncover molecular mediators, key pathogenic milestones and response to intervention at the metabolic level. The advent of precision imaging has yielded unprecedented insights into these processes. Quantitative imaging biomarkers such as magnetic resonance imaging (MRI), spectroscopy (MRS) and elastography (MRE) present robust, powerful tools with which to probe NAFLD metabolism and fibrogenesis non-invasively, in real time. Specific advantages of MRS include the ability to quantify static metabolite concentrations as well as dynamic substrate flux in vivo. Thus, a vast range of key metabolic events in the natural history of NAFLD can be explored using MRS. Here, we provide an overview of MRS for the clinician, as well as key pathways exploitable by MRS in vivo. Development, optimisation and validation of multinuclear MRS, in combination with other quantitative imaging techniques, may ultimately provide a robust, non-invasive alternative to liver biopsy for observational and longitudinal studies. Through enabling deeper insight into inflammatory and fibrogenic cascades, MRS may facilitate identification of novel therapeutic targets and clinically meaningful endpoints in NAFLD. Its widespread use in future could conceivably accelerate study design, data acquisition and availability of disease-modifying therapies at a population level.

## 1. Introduction

Non-alcoholic fatty liver disease (NAFLD) has rapidly emerged as a leading cause of chronic liver disease and liver transplantation in high-income economies [1]. Challenges to the development of effective therapy include the heterogenous, non-linear nature of disease progression, involvement of multiple organ systems and pathogenic bidirectionality, with NAFLD considered to be both the driver and the consequence of its many associations. Pathways to intervention will therefore rely on an integrated approach towards deeper understanding of the molecular mediators of whole-body insulin resistance, hepatocellular lipotoxicity, inflammation, oxidative stress and fibrogenesis and their combined role in the development and progression of NAFLD.

Whilst much is known about the causes and impact of NAFLD, research into its underlying metabolic and mechanistic processes is ongoing. This insight provides important foundations for developing clinical interventions to prevent or treat related diseases, and offers potential imaging biomarkers for diagnosis and longitudinal evaluation of treatment response. Magnetic resonance imaging (MRI) and spectroscopy (MRS) offer a unique non-invasive method of gaining understanding into both the physiological and metabolic progression of NAFLD with minimal ethical impact.

**Figure 1:**
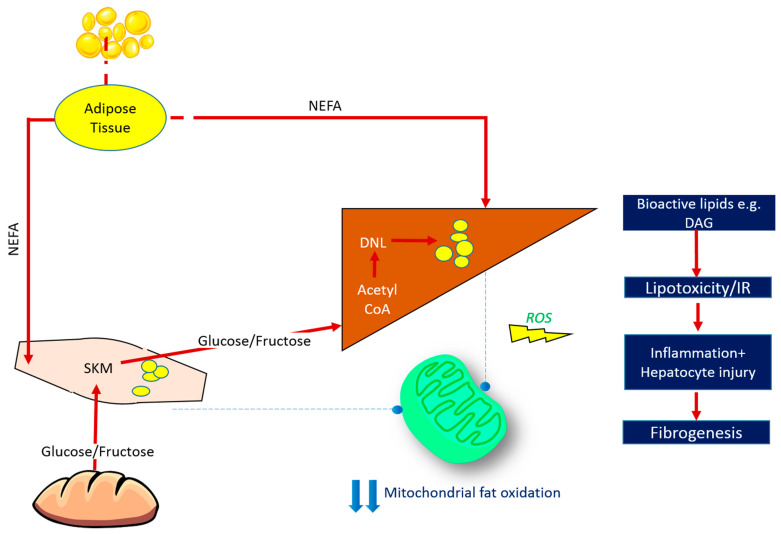
Fate of Ingested Macronutrients in States of Chronic Energy Surplus. Insulin resistance arises when nutrient storage pathways, which evolved to maximise efficient energy utilisation, are exposed to chronic energy surplus. Ectopic lipid accumulation in liver and skeletal muscle disrupts insulin signalling in these tissues, leading to reduced muscle glucose uptake and impaired hepatic glycogen synthesis. Muscle insulin resistance diverts ingested glucose to the liver, resulting in increased hepatic *de novo* lipogenesis and hyperlipidaemia. Increased liver fat stores are thus attributable to both increased free fatty acid (FFA) flux from insulin-resistant adipose tissue, and also diversion of dietary carbohydrate away from muscle glycogen synthesis and towards lipogenic pathways in the liver. The most biologically active form of liver fat is diacylglycerol, which is probably the moiety most directly responsible for impaired insulin signalling, with triglyceride being relatively metabolically inert. Reduced mitochondrial oxidative capacity in these tissues diminishes the ability of stored lipids to undergo oxidation, further exacerbating lipotoxicity and contributing toward oxidative stress. This culminates in necroinflammation and the onset of hepatic fibrogenesis. ROS = reactive oxygen species, SKM = skeletal muscle, NEFA = non-esterified fatty acids, DNL = de novo lipogenesis, DAG = diacylglycerol, IR = insulin resistance.

The concept of lipid-induced insulin resistance is key to the pathogenesis of NAFLD (Figure 1). In this paradigm, energy intake in excess of energy expenditure saturates subcutaneous and visceral adipose tissue lipid stores. Subsequent unrestricted lipolysis from adipose tissue leads to excess circulating free fatty acid (FFA), which is ultimately deposited in ectopic sites, including skeletal muscle and liver [2]. The body of mechanistic evidence available to date supports the hypothesis that accumulation of ectopic lipid—and in particular bioactive lipid species such as diacylglycerol (DAG)—triggers pathways that disrupt cellular insulin signalling, leading to reduced muscle glucose uptake and impaired suppression of hepatic glucose production [3]. Insulin resistance at the level of skeletal muscle diverts the fate of ingested carbohydrate away from muscle glycogen synthesis and towards lipogenic pathways in the liver (i.e., glucose is converted via hepatic *de novo* lipogenesis (DNL) into lipid species), further contributing to the development of NAFLD and dyslipidaemia. In the face of chronic energy surplus, the rate of lipid supply to insulin responsive tissues (i.e., liver and muscle) outstrips the rate of mitochondrial fatty acid oxidation (FAO). This favours the formation of reactive oxygen species and oxidative-stress with subsequent structural and functional mitochondrial damage [4,5,6]. Resultant accumulation of myocellular and hepatic lipid contributes to a vicious cycle of lipotoxicity, insulin resistance, and perturbed mitochondrial energy kinetics [7]. This ‘perfect storm’ of dysregulated metabolism gives rise to a pro-inflammatory, pro-fibrogenic milieu at the level of the liver, driving NAFLD progression.

The complexity of NAFLD mandates development of robust technologies capable of quantitatively evaluating diverse metabolic processes, in different organs, serially in real time in order to probe its pathophysiology and identify pathways amenable to therapeutic intervention. To this end, the advent of precision imaging has afforded a step change in the evaluation of NAFLD pathogenesis *in vivo*. Specifically, magnetic resonance spectroscopy (MRS) has proven to be a versatile and powerful tool in the quantification of liver and muscle fat, glycogen stores and phosphorus metabolites, each probing different dimensions in the complex evolution of metabolic liver disease.

This is of particular relevance to the study of hepatic and whole-body insulin resistance, given the ability to simultaneously quantify lipid and glucose metabolism in liver and muscle, as well as energy homeostasis using ^1^H-MRS, ^13^C MRS, and ^31^P-MRS respectively. Other MR modalities, including magnetic resonance imaging (MRI) and magnetic resonance elastography (MRE), afford unique insights into liver structure and stiffness, respectively. A combined approach, employing MRI, MRS, and MRE can therefore yield detailed information on liver anatomy, metabolic function, fibrosis burden, and the role of the liver-muscle axis in driving a NAFLD phenotype.

In this review, we discuss the various applications of MRS in experimental hepatology with a focus on yielding insights into NAFLD pathogenesis. In particular, we discuss the scope of non-proton MR spectroscopy and specific experimental techniques exploitable for mapping key metabolic pathways and substrate flux in vivo. Further, we speculate on the role of ultra-high field strength MR scanners in a clinical sphere, both as technologies with which to probe the natural history of NAFLD and as novel endpoints with which to evaluate response to intervention in clinical trials. While routine use of MRS in the clinical evaluation of NAFLD is not yet practiced, pathways to clinical impact lie in the identification of key imaging biomarkers which correlate closely with pathogenic milestones and response to intervention.

## 2. Principles of MR Spectroscopy

MRS remains predominantly a research tool requiring significant expertise over and above MRI. However, emerging novel MRS methodologies offer insight into key mechanistic features of NAFLD in a non-invasive way that can be applied longitudinally throughout the disease progression. MRS works alongside MRI to gain a greater depth of insight as it is not limited to molecules containing ^1^H, namely water and fat. As such, other metabolite concentrations and flux rates indicative of mitochondrial function and oxidative stress can be measured (e.g., glycogen, ATP, glutathione). In addition, the structure of lipid molecules stored in hepatocytes can be probed. As these technologies become more established there is clear potential for translation into a clinical setting which offers significant advantages over biopsy. Current limitations of MRS over MRI include the use of non-standard equipment and increased expertise which also affects the overall costs [8]. If findings from current research prove useful for diagnostic and therapeutic purposes, then these limitations will become more comparable to MRI.

**Figure 2:**
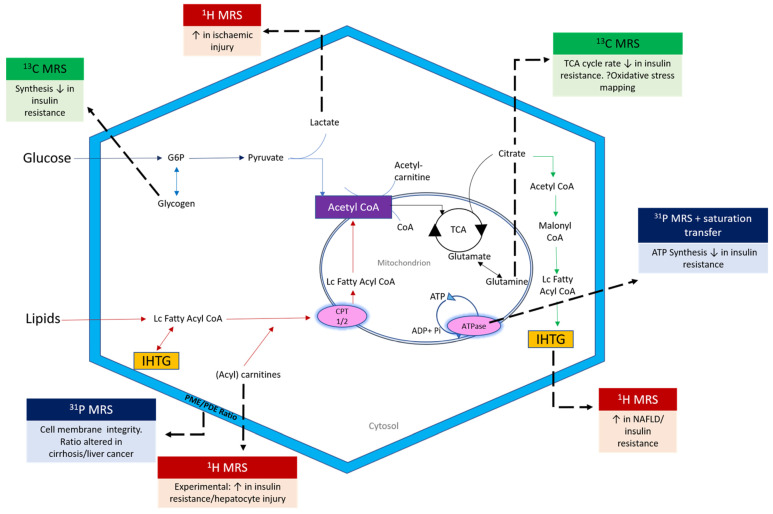
Scope of multinuclear MR Spectroscopy in the interrogation of metabolic liver disease. LC = Long chain; G6P = glucose 6 phosphate; CPT = carnitine palmitoyl transferase; PME = phosphomonoester; PDE = phosphodiester; Pi = inorganic phosphate.

Both MRI and MRS measures of liver fat have been shown to correlate closely to histological estimates of liver fat in NAFLD populations, as well as to each other [9]. While MRI allows detailed insight into tissue structure and spatial location, MR spectroscopy provides chemical information about specific metabolite concentrations and dynamics within a tissue pool of interest. More complex sequences can also be employed to determine the rates of metabolic activity. The role of MR spectroscopy has vastly expanded in recent years, to include detailed interrogation of liver and muscle metabolism (Figure 2).

**Figure 3:**
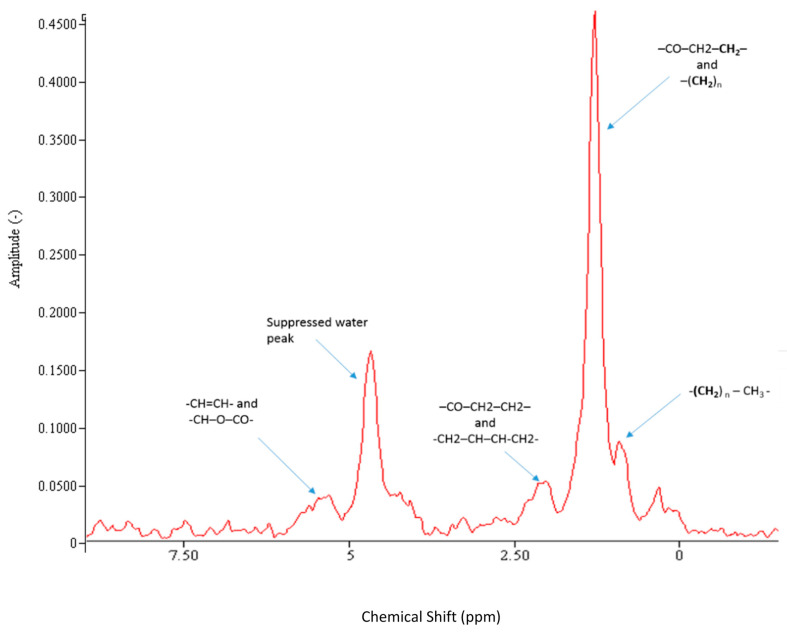
Liver ^1^H MR Spectrum (3T) with water suppression acquired in vivo from an individual with NAFLD. The different fat peaks resolvable by spectroscopy at 3T are shown. CH=CH (olefinic acid), CH-O-CO (glycerol), CO-CH_2_-CH_2_ (α carboxyl), CH_2_-CH=CH-CH_2_ (α olefinic), CO-CH_2_-CH_2_ (β carboxyl), (CH_2_)n (methylene), (CH2)n-CH_3_ (methyl). Spectral resolution at 3T is excellent, allowing for accurate fitting and quantification of IHTG. The fat fraction is derived from dividing the fat content by the combination of fat and water content.

The basic physical principles underpinning MRI and MRS are identical. Differences in output and scope are highlighted in Table 1. For conventional MRI, a magnetic field gradient is introduced so that hydrogen signals from water and fat can be spatially located within a large field of view. By contrast, MRS focusses on a smaller region of interest and acquires a signal with much greater chemical resolution. This signal can be transformed into a spectrum that is a plot of signal frequency (*x* axis) versus intensity (*y* axis) as shown in Figure 3. Different metabolites occupy multiple unique frequencies along the MR visible spectrum. For example, in the proton (^1^H) MR spectrum, the predominant signal is from water (4.7 ppm), whereas protons from fat align at different distinct frequencies (e.g., CH_2_ peak at 1.3 ppm), enabling separation of different metabolites of interest [10]. The total signal from a specific metabolite is measured by integrating the area under the spectral peaks and compared to some known reference molecule (e.g., water) to determine concentration.

In MRS, the frequency of nuclei is conventionally converted to parts per million (ppm) relative to tetramethylsilane (TMS) so that it remains the same for a given metabolite across different strength scanners. Different biochemical signatures in healthy versus diseased tissue can help to identify and localise disease at an early stage in its natural history. In order to elucidate relevant information, a number of scanning techniques are required that are not used in MRI. These include localisation of a specific region of interest, which typically uses field gradients alongside refocusing pulses, and water suppression so that other less dominant metabolites can be measured accurately. Over recent decades many new techniques have been developed to enhance the signal acquired and to measure the metabolic activity, such as saturation transfer.

Whilst MRI works exclusively with signal from ^1^H nuclei (due to its high abundance and MR visibility), MRS can be used to acquire signal from other nuclei, such as ^13^C and ^31^P, which are less prevalent. Dedicated coils are required that are tuned to the frequency of these nuclei, and alternative sequences will often be used to enhance the signal.

## 3. Applications of MRS in Metabolic Liver Disease

Table 2 summarises the metabolic processes that constitute key milestones in NAFLD progression. Multinuclear MR spectroscopy, employing dedicated coils, offers an exciting and novel window into the metabolism of hydrogen, carbon and phosphorus-based compounds. This enables researchers to probe multiple dimensions of metabolic liver disease contemporaneously, including lipotoxicity, oxidative stress, β-oxidation, cell membrane integrity and bioenergetics (Figure 2).

### 3.1. ^1^H MRS

Proton (^1^H) MR spectroscopy is the most widely available technique and can be applied in experimental hepatology to accurately quantify intrahepatic triglyceride (IHTG). MRS-derived measures of IHTG are increasingly being used as important outcome measures in observational studies and longitudinal clinical trials involving dietary or lifestyle interventions in NAFLD. These techniques have been validated against conventional histology and are considered gold-standard for non-invasive assessment of hepatic steatosis [11].

#### 3.1.1. Probing the Liver-Muscle Axis

**Figure 4:**
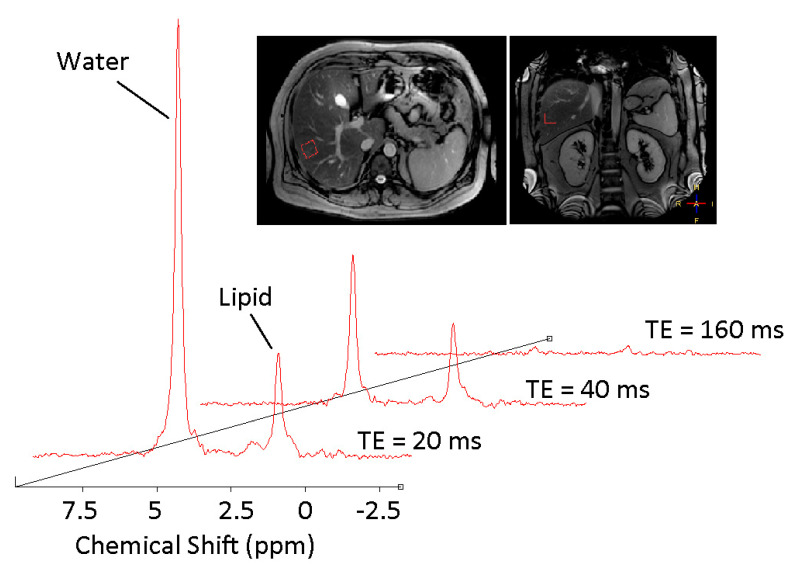
Typical ^1^H MR spectra from an individual with NAFLD. The fat fraction is derived from dividing the fat content by the combination of fat and water content. Note that spectra are taken at four different echo times and averaged. Scout images demonstrate localisation within the right lobe of the liver, ensuring the region of interest is placed away from major blood vessels and subcutaneous tissue.

Ectopic fat deposition is a key pathological milestone in the development of hepatic and whole-body insulin resistance [12]. Probing the mechanisms underpinning ectopic fat deposition through quantitative evaluation of both liver and muscle lipid content in real time (Figure 3 and Figure 4) yields unique insights into the pathogenesis of systemic lipotoxicity. ^1^H MRS permits serial changes in the accumulated fat to be probed, and can be used in combination with other techniques, such as the euglycaemic hyperinsulinaemic clamp, to quantify hepatic, muscle and whole-body insulin resistance.

Lipid accumulation in skeletal muscle has been strongly implicated as an early event in the development of NAFLD and whole-body insulin resistance [13]. A series of MRS-based studies in insulin-sensitive and matched insulin resistant phenotypes support the hypothesis that muscular lipid accumulation impairs local insulin signalling, thus attenuating insulin-mediated glucose disposal and glycogen synthesis within the muscle. For example, Petersen and colleagues explored the response to two high carbohydrate meals of young, healthy, lean insulin-resistant subjects (*n* = 12) compared with age, BMI, activity and fat mass-matched insulin sensitive subjects (*n* = 12) [14]. Utilising ^1^H-MRS and ^13^C-MRS, the group studied muscle and liver triglyceride and glycogen synthesis, respectively. Through measuring incorporation of deuterated water into plasma triglycerides, the group quantitatively determined postprandial fractional hepatic *de novo* lipogenesis. Novel findings included a 60% reduction in net muscle glycogen synthesis following meals in the insulin-resistant cohort, together with a 2.5-fold net increase in hepatic triglyceride synthesis and a 2.2 fold increase in postprandial fractional hepatic *de novo* lipogenesis in these individuals. These data suggest that selective skeletal muscle insulin resistance alters the pattern of carbohydrate energy storage, favouring development of NAFLD and dyslipidaemia, and may be the earliest harbinger of the metabolic syndrome.

**Figure 5:**
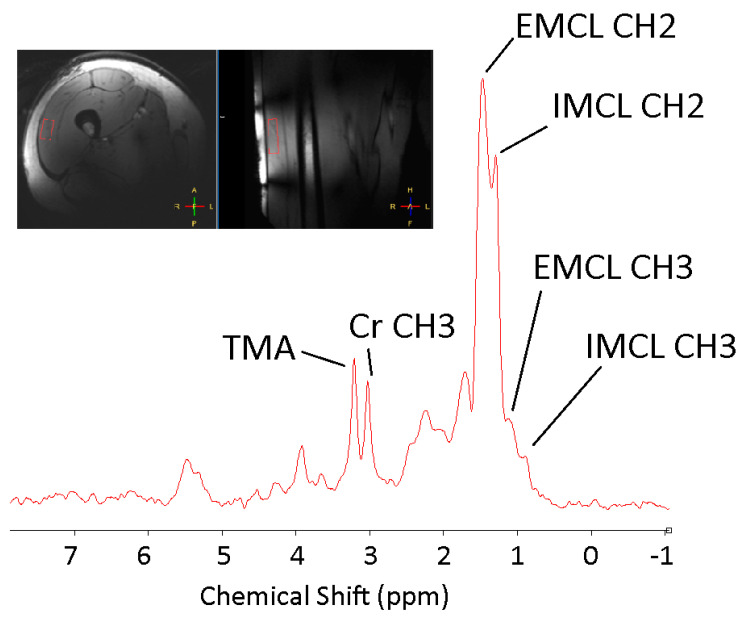
^1^H MRS spectrum acquired from the vastus lateralis muscle at 7T. At a high magnetic field (7T) resolution permits separation of peaks representing intramyocellular (IMCL) and extramyocellular (EMCL) lipids (CH_2_ groups at 1.3 and 1.5 ppm respectively, CH_3_ groups at 0.9 and 1.1 ppm, respectively, Creatine (3.03 ppm) and the trimethylamine group (TMA), comprising choline, carnitine and acetylcarnitine.

Within muscle tissue, lipid moieties either accumulate as droplets within myocyte cytoplasm—so-called intramyocellular lipid (IMCL), or are deposited along the fibres between muscle cells as interstitial triglycerides (extramyocellular lipids [EMCLs]) (Figure 4). The metabolic consequences of these fractions differ: EMCL is considered metabolically inert, whereas IMCL, if supply exceeds expenditure, directly impacts upon cellular insulin signalling [15]. MRS offers a non-invasive method to differentiate IMCL from EMCL, as both fractions are resolvable along the ^1^H MR spectrum, with excellent resolution at higher field strengths (Figure 5). The notion that IMCL accumulation has a potentially causal role in the development of systemic insulin resistance has gained increasing traction in recent years and has been consistently reported as a strong correlate of insulin resistance in non-diabetic individuals, including young, lean offspring of individuals living with type 2 diabetes (T2DM) [16,17].

#### 3.1.2. Evaluating Response to Intervention

The effects of alterations in macronutrient composition on liver fat have been investigated by harnessing ^1^H MRS to evaluate response to short-term dietary interventions. Markova et al. (2017) conducted a study in 37 individuals with type 2 diabetes on an isocaloric diet plan for 6 weeks, (30% protein, 40% carbohydrate, 30% fat). Eighteen patients were allocated to a diet high in animal protein and 19 were allocated to a diet high in plant protein. Euglycaemic hyperinsulinaemic clamp studies were conducted at baseline and post-intervention to determine the metabolic effects of each diet. After 6 weeks, the group concluded that both animal and plant proteins reduced IHTG by 36–48% with improvements in insulin sensitivity in both groups [18].

Another study used ^1^H MRS to evaluate IHTG at baseline and after 12 weeks of isocaloric weight-maintaining dietary interventions enriched with either monounsaturated fatty acids (MUFA) or fibre, as compared with control dietary protocols in a prediabetic NAFLD population (*n* = 43). In this analysis, MUFA-enriched diet was associated with a significant reduction in liver fat (relative reduction 18 ± 3%, *p* < 0.01) compared to control, with improved hepatic and whole-body insulin sensitivity as assessed by a labelled [6, 6 ^2^H_2_] oral glucose tolerance test [19].

The sensitivity of MRS in detecting changes in IHTG has been exploited to enable study of acute postprandial effects of specific dietary patterns. For example, Browning et al. (2011) demonstrated that just 2 weeks of low-carbohydrate diet (<20 g/day) resulted in a significant reduction in IHTG (−55 ± 14%) versus general calorie restriction alone (−28 ± 23%) with similar weight loss in both groups [20].

In another study, nine healthy subjects were studied before and after a single high fat meal with ^1^H MRS at high-field strength (3T) to evaluate both IHTG and intramyocellular lipid content. The authors demonstrated that a single energy dense, high fat meal was sufficient to induce ectopic lipid accumulation, with a 20% increase in IHTG stores at 3 h post-meal [21]. Muscle lipid content, in contrast, was not altered acutely, suggesting that muscle tissue may be less sensitive to short-term changes in circulating triacylglycerol concentrations.

As well as probing fat quantity, MRS provides unique insight into qualitative fat signatures, enabling determination of saturated versus unsaturated lipid stores. This is of particular interest in NAFLD, which is characterised by the depletion of polyunsaturated lipid stores [22]. In one study, the effect of short-term (7 days) aerobic exercise on quantity and quality of intrahepatic lipid was explored in obese individuals (*n* = 17) with NAFLD. Results confirmed that short-term exercise interventions increased intrahepatic polyunsaturated lipids. These changes were associated with increased circulating adiponectin, implying a favourable metabolic response [23]. In a recent cross-sectional study, Roumans et al. (2020) optimised hepatic ^1^H MRS at 3T to discriminate saturated fatty acids (SFA), as well as MUFA and polyunsaturated fatty acids (PUFA) fractions in healthy individuals across a wide range of intrahepatic fat [24]. The group demonstrated that rates of *de novo* lipogenesis (DNL) correlated positively with the fraction of hepatic SFA (*p* = 0.047), and confirmed a strong negative correlation between DNL and MUFA fraction (*p* = 0.003). Furthermore, hepatic SFA fraction correlated inversely with hepatic insulin sensitivity (*p* = 0.002). Taken together, these findings highlight the power of ^1^H MRS-based lipid composition analysis in mapping a specific metabolic phenotype at heightened risk of metabolic complications in NAFLD, and potentially offer an exciting avenue for the non-invasive evaluation of hepatic *de novo* lipogenesis.

### 3.2. ^13^C MR Spectroscopy

The liver acts as an intermediary hub for both lipid and glucose metabolism. ^13^C MR spectroscopy adds enormous value in the interrogation of metabolic liver disease, enabling net glycogen synthesis, glycogenolysis and gluconeogenesis to be quantified, both at baseline and in response to various stimuli such as dietary and exercise interventions [25].

**Figure 6:**
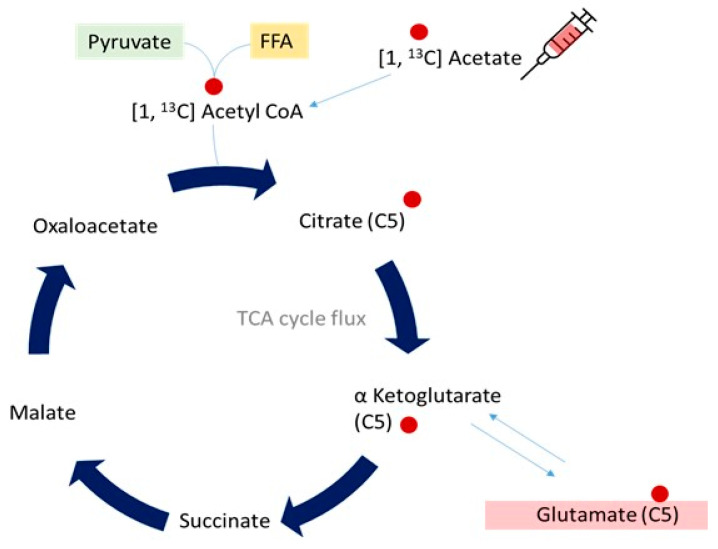
Schematic diagram demonstrating the fate of infused ^13^C labelled acetate upon incorporation into hepatic metabolism. Following degradation to ^13^C Acetyl CoA, fractional enrichment of hepatic ^13^C glutamate (with the ^13^C nucleus at the C5 position) is measurable on the MR visible spectrum, enabling calculation of TCA cycle flux.

Spectral resolution of carbon nuclei in the liver is hindered by low natural abundance of the ^13^C isotope, which is MR visible (1.1% of total carbon, the rest being ^12^C which is not MR visible) [26]. Methods commonly employed to address this include long acquisition times and large regions of interest, with tracer labelling strategies in experimental protocols to enhance abundance of ^13^C nuclei. In human experiments, infusion or ingestion of ^13^C labelled substrates (e.g., acetate or glycine) can be exploited as a powerful tool with which to determine various facets of metabolism, from tricarboxylic acid (TCA) cycle activity to cellular redox kinetics. This technique relies on the principle that increases in ^13^C signals following administration of enriched substrate reflect incorporation of that substrate into the metabolic pathway of interest [27]. An example schematic of isotopic enrichment of ^13^C nuclei with labelled substrate ([1, ^13^C] acetate) incorporation into intermediates in the TCA cycle is shown in Figure 6. The rate of signal increase on ^13^C MRS then reflects rate of TCA cycle activity that can be mathematically modelled in both healthy and metabolically challenged phenotypes.

#### 3.2.1. Mitochondrial Oxidative Metabolism

Dysregulated hepatic oxidative metabolism is widely considered to contribute to NAFLD pathogenesis. Disturbed mitochondrial structure, function, or both leads to impaired energy production, oxidative stress and release of reactive oxygen species (ROS), inflicting a pro-inflammatory milieu at the level of the liver. Although MRI and mass spectrometry techniques are well established, these provide limited information regarding mitochondrial metabolism in NAFLD as they measure static metabolite concentrations. Metabolite kinetics, measurable by capturing flux rates through key enzymatic pathways, yield deeper mechanistic insight into NAFLD pathogenesis. MRS is ideally suited to this endeavour, as repeated perturbations in metabolite synthesis or clearance following intervention can be quantitated at serial time points. Using this framework, strategies have been developed to directly determine rates of hepatic mitochondrial oxidation as well as oxidative stress reactions noninvasively.

Befroy and colleagues recently developed a novel MRS-based protocol to quantify hepatic mitochondrial oxidative metabolism utilising localised in vivo ^13^C-MRS. In this strategy, intravenous ^13^C- labelled acetate (a stable isotope tracer) was administered to healthy volunteers to become incorporated, via its intermediate ^13^C-acetyl-CoA, into the TCA cycle in the liver. The rate of ^13^C enrichment of hepatic glutamate (its principal metabolite) can then be tracked as glutamate is MR-visible in ^13^C-spectra at the C1 and C5 positions in the liver [28]. These parameters are valuable, as the TCA cycle forms the final common pathway for both carbohydrate and lipid metabolism.

A subsequent study incorporating this method in patients with NAFLD concluded, surprisingly, that rates of mitochondrial oxidative metabolism were not diminished in subjects with NAFL (as defined by IHTG > 4%) compared with control populations [29]. In this study, Petersen and colleagues utilised rate of ^13^C enrichment of hepatic glutamate to measure mitochondrial oxidation flux and ^13^C enrichment of hepatic alanine as an index of pyruvate cycling. However, the study specifically selected individuals without insulin resistance, who had isolated steatosis on MRS but no evidence of NASH or fibrosis. Further studies in histologically characterised NAFLD populations are needed to determine whether mitochondrial TCA activity is compromised in NASH versus NAFL, and thus to determine whether measuring hepatic oxidative metabolism could serve as an accurate imaging biomarker to discriminate individuals at risk of disease progression.

#### 3.2.2. Oxidative Stress

Mitochondrial oxidative stress may be probed non-invasively using ^13^C MRS labelling strategies. The ability of hepatocytes to maintain a reduced intracellular environment protects against oxidative stress and the associated onslaught of necroinflammation and fibrogenesis that underpin NAFLD progression. The prevailing endogenous hepatic antioxidant is glutathione, a tripeptide synthesised from glycine, glutamate and cysteine. Decreased glutathione synthesis is associated with impaired hepatocellular redox defences, leaving liver tissue vulnerable to free radical-induced damage. Glutathione turnover has thus been the focus of recent attention as a potentially useful biomarker for monitoring hepatic oxidative stress in NAFLD. By extension, the ability to track glutathione flux in vivo could yield a clinically useful quantitative biomarker for mapping disease activity in NAFLD.

Skamarauskas et al. (2014) developed a dynamic ^13^C-MRS labelling strategy to measure the flux through glutathione (GSH) synthesis pathways in vivo [30]. This approach was demonstrated in human volunteers in addition to rodent models of acute and chronic oxidative stress. Briefly, ^13^C-labelled glycine was administered via infusion in Sprague Dawley rats to measure hepatic glutathione flux in response to acute (CCl_4_ induced injury) and chronic (high fat, high carbohydrate diet for 8 weeks) oxidative insults. Associated perturbations in glutathione metabolism were measured in a 7T MR scanner. In acute CCl_4_-induced oxidative stress, preclinical studies demonstrated a 54% elevation of GSH content and a 31% increase in the GSH synthesis pathway (as measured by ^13^C label incorporation of hepatic glutathione), after 12 h, indicating adaptive upregulation of glutathione utilisation in response to acute oxidative stress.

Conversely, the chronic model of oxidative stress revealed glutathione depletion in an early NASH phenotype after 8 weeks of a high-fat high carbohydrate feeding. This novel experimental approach was successfully translated in human volunteers, with oral ingestion of ^13^C labelled glycine detectable on ^13^C MRS at 2, 4, 6 and 8 h post-ingestion. Exciting clinical applications include a non-invasive approach for direct quantification of hepatic glutathione fluxes in response to intervention and use of such measures as a biomarker for NAFLD staging. Indeed, pharmacological interventions which increase glutathione synthesis in NAFLD may provide a therapeutic target with which to attenuate development of steatohepatitis.

### 3.3. ^31^P MRS

While capturing hepatic mitochondrial metabolism using non-invasive imaging technology is possible using labelled ^13^C-MRS strategies, the utility of localised ^31^P MRS in quantitating hepatic ATP production has gained attention in recent years as the phosphorus isotope is present in high natural abundance.

**Figure 7:**
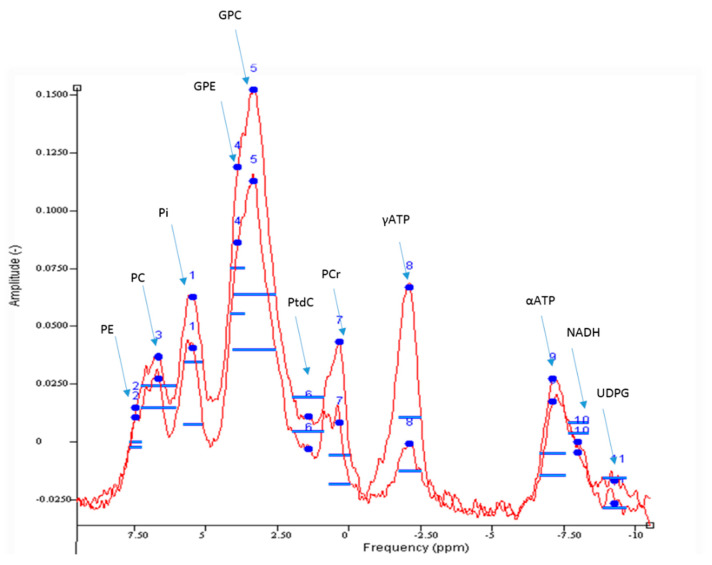
Example ^31^P MR Spectrum acquired from the liver at 3T. Good spectral resolution enables quantification of phosphorus metabolites including adenosine triphosphate (ATP), phosphomonoesters (phosphocholine, PC and phosphoethanolamine, PE), phosphodiesters (glycerophosphocholine, GPC and glycerophosphoethanolamine, GPE), nicotinamide adenine dinucleotide (NADH), uridine diphosphate glucose (UDPG) and phosphatidylcholine (PtdC).

^31^P-MRS enables quantification of inorganic phosphorus compounds within the liver (Figure 7). These include phosphomonoesters (PME) and phosphodiesters (PDE), as well as inorganic phosphate (Pi) and adenosine triphosphate (ATP). Together, PME and PDE provide a surrogate measure of cell membrane integrity and turnover, which can correlate with histological fibrosis burden in chronic liver disease [31]. Hepatic ATP and Pi levels are also detectable in the ^31^P spectra and their absolute quantification can provide insight into hepatic energy metabolism. This is of particular relevance in NAFLD, as progressive depletion in hepatic ATP stores are observed in individuals with features of the metabolic syndrome, including obesity and type 2 diabetes, reflecting impaired energy homeostasis associated with insulin resistance [32]. Thus, noninvasive monitoring of hepatic ATP stores could serve as a quantitative imaging biomarker for evaluating key pathogenic milestones in NAFLD.

#### 3.3.1. Hepatic ATP Reserves

**Figure 8:**
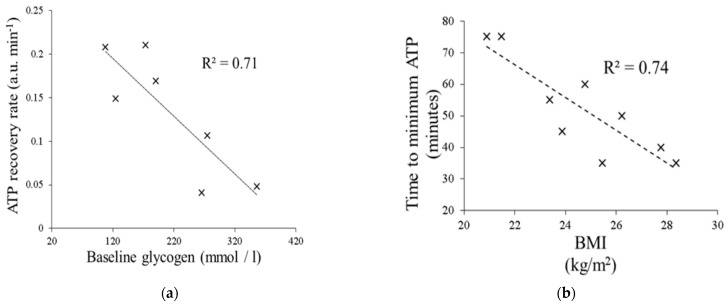
(**a**) Time to ATP recovery is inversely proportional to hepatic glycogen content. (**b**) Increasing BMI is associated with a shorter time to hepatic ATP depletion.

We have investigated the effects of an oral fructose challenge on hepatic ATP reserves (as measured by ^31^P-MRS) and time to ATP depletion in healthy adult males across a spectrum of body mass index. Fructose is well-known to deplete hepatic ATP reserves through its rapid fructokinase-induced phosphorylation and lack of negative feedback regulation [33]. Through administration of a 75 g oral fructose challenge followed by immediate ^31^P-MRS, we demonstrated a negative correlation between time to minimum hepatic ATP levels and BMI, suggesting impaired hepatic energy metabolism with progressive increases in BMI (Figure 8) [34]. Further, rates of ATP recovery in the liver were negatively correlated with hepatic glycogen content as assessed using ^13^C MRS. Together, these data suggest that quantitative assessment of hepatic ATP reserves, and time to ATP recovery following dietary challenge, could serve as a dynamic ‘stress’ test for the metabolically challenged liver.

#### 3.3.2. Saturation Transfer Experiment

**Figure 9:**
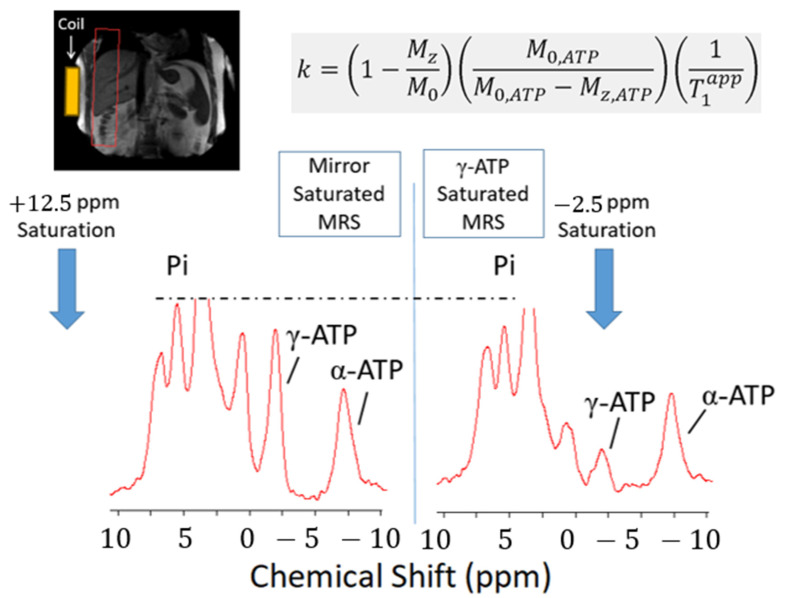
Saturation transfer technique in ^31^P MRS. A frequency selective saturation pulse is applied to the gamma ATP peak in the phosphorus spectrum, with corresponding saturation… With this technique, ATP kinetics can be mathematically modelled utilising a first-order differential equation together with a rate constant, to calculate the forward rates of ATP synthesis in the liver.

Use of saturation transfer enables the rate of hepatic ATP turnover to be measured through calculation of forward exchange flux in the Pi-to-ATP reaction, catalysed by the enzyme ATP synthase (i.e., ADP + Pi ⇌ ATP, Figure 9). This exchange rate constant (*k*) provides a surrogate, rather than direct, measure of mitochondrial oxidative metabolism. Nevertheless, high natural abundance of phosphorus metabolites in vivo negates the requirement for tracer labelling techniques, enhancing clinical applicability of this method in the assessment of hepatic energy metabolism. Schmid and colleagues (2011) first demonstrated that ATP production in T2DM individuals without clinical liver disease is lower than that of matched control subjects without T2DM, supporting a close association between insulin resistance and perturbed hepatic energy metabolism [35].

7T scanners permit reduction in acquisition time for saturation transfer experiments, from 2 hours to approximately 20-25 minutes. In a cross-sectional study assessing reproducibility of localised in vivo ^31^P-MRS of the liver at 7T, Valkovic and colleagues determined differential ATP turnover between histological subtypes of NAFLD. In comparison with healthy volunteers, the group demonstrated that individuals with NASH had significantly lower ATP production (k = 0.17 ± 0.04 Mm/s versus 0.31 ± 0.03 Mm/s, *p* < 0.01). Those with isolated steatosis (NAFL), however, had baseline Pi to ATP exchange rates very similar to healthy controls (k = 0.30 ± 0.05 Mm/s) [36]. More recently, Traussnigg and colleagues employed saturation transfer ^31^P-MRS at 7T in a prospective clinical trial to differentiate ATP flux in patients with biopsy-proven isolated steatosis (NAFL) and NASH. The group confirmed that rate of ATP synthesis is significantly decreased in NASH compared with NAFL (0.21 ± 0.08 Mm/s versus 0.38 ± 0.08 Mm/s, *p* = 0.003). Taken together, these data highlight disturbed energy metabolism in NASH compared with NAFL and support the hypothesis that hepatic mitochondrial dysfunction may be an important determinant of this distinction [37].

#### 3.3.3. Other Uses of ^31^P MRS in the Non-Invasive Stratification of NAFLD

Non-invasive assessment of hepatocellular metabolism using ^31^P MRS includes acquisition of hepatic PME, PDE, and TP levels and calculation of their ratios. PME and PDE metabolites broadly reflect cell membrane turnover and have been reported as markers of endoplasmic reticular stress [38]. The ratio of hepatic PME and PDE metabolites may provide a surrogate measure of inflammation and fibrosis burden in diffuse liver diseases. This concept was initially applied in Hepatitis C-related liver disease, where ^31^P-MRS measures of fibrosis were compared against liver biopsy, demonstrating a strong positive correlation between PME/PDE ratio and histological disease severity [39]. Similar findings have been replicated in NAFLD populations [40]. Whether changes in the PME/PDE ratio reflect fibrosis regression *per se* is unknown. The evolution and regression of fibrosis are broadly considered to be time-consuming processes [41]. Rapid reductions in liver stiffness (as assessed by transient elastography) following viral eradication in Hepatitis C may be more reflective of a reduction in inflammation [42,43]. Thus, MRS may be valuable in evaluating disease progression, but the significance of any change in PME/PDE ratio upon treatment and how this correlates to histological fibrosis burden, is unknown.

In a recent cross-sectional study, Abrigo and colleagues (2014) assessed diagnostic performance of ^31^P MRS (3T) for biochemical differentiation of NAFL versus NASH in a cohort of biopsy-proven patients. The group reported a decreased total nucleotide triphosphate (NTP)/TP ratio in NASH patients (*p* = 0.004) versus those with isolated steatosis (*p* = 0.047), indicating disturbed energy homeostasis and highlighting the utility of this measure as a discriminating parameter with a 91% sensitivity and 91% specificity for NASH [44]. In addition, the group reported elevated PDE in NAFLD and NASH, possibly reflecting increased cell membrane degradation and endoplasmic reticulum (ER) stress in the context of lipotoxicity and steatosis. Glycerophosphocholine (GPC) levels were negatively correlated with fibrosis (ƥ = −0.361, *p* < 0.001), indicating that GPC could serve as a non-invasive fibrosis marker. This is supported by other reports of a stepwise decrease in GPC/ (PME+PDE) from NAFL to NASH and cirrhosis [33].

Ultimately, further studies are required to determine the clinical utility of non-invasive stratification of NAFLD subtypes, both clinically and histologically. However, the landscape appears promising for the use of ^31^P MRS in NAFLD. In combination with ^1^H and ^13^C MRS, this methodology can accurately map, and determine the interaction between hepatic fat, glucose and energy homeostasis in the metabolically challenged liver.

##### Limitations

While MRS offers promising insights into the mechanistic drivers of NAFLD, NASH and fibrogenesis, important limitations preclude its routine incorporation in clinical practice. First, MRS determines metabolite concentrations in a specific voxel of tissue. Typically, a voxel may be 20 × 20 × 20 mm^3^, with MRS probing metabolites in that region. While significantly greater than the volume of tissue obtained from liver biopsy, this nevertheless introduces potential for sampling error, particularly in diffuse liver diseases such as NAFLD, where there is heterogenous distribution of fat. A further challenge to widespread uptake of MRS is the specialised software required for post-processing of spectra, as well as specific hardware (i.e., surface coils and interface hardware) involved in multinuclear MRS, and their associated cost. Finally, MRS data acquisition and interpretation require significant expertise which is not currently incorporated into clinical GI radiology training outside of specialised centres. Such training may evolve with the demonstration of clinical applicability, the landscape of available technology and need for longitudinal evaluation of disease-modifying therapies non-invasively.

## 4. Conclusions

The slow nature of disease progression in NAFLD, heterogeneity of therapeutic targets and well-established limitations of serial liver biopsy to evaluate the effects of intervention remain obstacles to the characterisation of pathogenic milestones and development of effective therapies. MRS could prove a useful tool in experimental hepatology to noninvasively probe key metabolic pathways and substrate fluxes integral to inflammation and fibrogenesis in NAFLD. In time, and if shown to correlate robustly with histological endpoints, MRS could be used to stratify patients at risk of disease progression, for example to prioritise such individuals for disease-modifying treatment. Serial evaluation of treatment response through MRS is also possible.

Harnessing these techniques in observational studies and longitudinal trials will help overcome several of the burdens currently associated with the development of effective therapies, including the identification of therapeutic targets, development of clinically meaningful metabolic endpoints and evaluation of the acute and long-term effects of dietary, lifestyle and pharmacological interventions. In summary, MRS presents a viable, potentially cost-effective tool, with clinical applications in NAFLD. It may complement liver biopsy in the stratification of disease burden, yield mechanistic insight into drivers of NAFLD progression and provide dynamic metabolic information in vivo.

## Figures and Tables

**Table 1:** Differences between MRI and MRS in NAFLD application.

	MRI	MRS
Principle Output	Anatomic	Metabolic
Use of signal	Spatial position of water and fat	Chemical composition of tissue
Region Covered	Structure of multiple tissues	Tissue specific region of interest One (Single voxel spectroscopy) or multiple (chemical shift imaging) voxels
Nuclei of interest	^1^H signals from fat and water	^1^H, ^13^C, ^31^P, ^23^Na, ^19^F
Data Acquisition	Images	1D/2D Spectra
Advantages	Standard equipment used Known sequences Easily translatable visualisations Shorter scan times Multi-organ single-shot images Non-invasive	Organ specific Metabolite concentration Lipid composition analysis Measure dynamic processes Increased sensitivity on fat Non-invasive
Limitations	Cost (compared to biological sampling) Precision of fat fraction measurement Only images fat and water	Non-standard equipment required Expertise/training necessary Cost (compared to MRI)

**Table 2:** Non-invasive quantitative biomarkers with which to probe NAFLD, NASH and fibrosis burden. NFS = NAFLD fibrosis score; FIB-4 = fibrosis-4 index; ELF = extended liver fibrosis panel; ProC3 = pro-collagen C3; ALT= alanine aminotransferase; TE = transient elastography; CAP = controlled attenuation parameter USS = ultrasound; HOMA-IR = homeostasis model of insulin resistance; HbA1c = haemoglobin A1c; TCA = tricarboxylic acid cycle; ATP = adenosine triphosphate; PDFF = proton density fat fraction.

Pathogenic Pathway	Biological Process	Precision Imaging Endpoints	Other Endpoints
Metabolic Inflexibility	Insulin Resistance	^13^C MRS (↓ postprandial net hepatic glycogen synthesis)	Dual Step Euglycaemic Hyperinsulinaemic Clamp to assess hepatic and peripheral insulin sensitivity Oral glucose tolerance test Wet biomarkers: Adiponectin, HbA1c, HOMA-IR
	Liver fat Quantity (%)	MRI-PDFF, ^1^H MRS	TE with CAP, USS
	Liver fat Quality	^1^H MRS	
Inflammation	Oxidative Stress	^13^C MRS (glutathione flux)	
	Impaired Energy Kinetics	^31^P MRS (ATP flux) ^13^C MRS (TCA cycle flux and β-oxidation)	
	Steatohepatitis		Risk Prediction Tools: OxNASH, NASH resolution score Wet biomarkers: ALT
Collagen deposition	Fibrosis burden	Magnetic Resonance Elastography (MRE)	Wet biomarkers: ELF, ProC3
		^31^P MRS (PME/PDE ratios)	Risk prediction tools: FIB-4, NFS
